# Flow Cytometric Test with Eosin-5-Maleimide for a Diagnosis of Hereditary Spherocytosis in a Newborn

**DOI:** 10.1155/2019/5925731

**Published:** 2019-05-07

**Authors:** Kanda Fanhchaksai, Suphara Manowong, Rungrote Natesirinilkul, Lalita Sathitsamitphong, Pimlak Charoenkwan

**Affiliations:** ^1^Research Cluster of Thalassemia and Red Blood Cell Disorders, Faculty of Medicine, Chiang Mai University, Chiang Mai 50200, Thailand; ^2^Department of Pediatrics, Faculty of Medicine, Chiang Mai University, Chiang Mai 50200, Thailand

## Abstract

A term male newborn born to a mother who had hereditary spherocytosis presented with neonatal jaundice at 20 hours of life. Complete blood count showed hemoglobin 17.1 g/dL, MCV 104.2 fL, MCH 32.9 pg, and MCHC 31.6 g/dL. The patient had indirect hyperbilirubinemia requiring phototherapy. The maximum total bilirubin level was 12.15 mg/dL at 20 hours of life. Peripheral blood smear revealed spherocytes, crenated red cells, and polychromasia. A flow cytometric test with eosin-5-maleimide- (EMA-) labeled RBC was performed in the patient and parents. The fluorescence histograms of EMA-labeled RBC from the patient and mother were shifted to the left, and the fluorescence ratio when compared with normal was 0.69 and 0.84, respectively. The flow cytometric test with EMA is useful in supporting the diagnosis of hereditary spherocytosis during newborn period.

## 1. Introduction

Hereditary spherocytosis (HS) is an inherited red blood cell (RBC) membrane disorder resulted from deficiencies of cytoskeletal membrane protein involving in the vertical connection between the phospholipid bilayer and structural proteins of the cell membrane [1]. The clinical severity varies from mild to severe anemia [1]. Newborns with HS may present with indirect hyperbilirubinemia requiring phototherapy or exchange transfusion.

HS is generally diagnosed by the significant presence of spherocytes in peripheral blood smear. The diagnosis can be confirmed by osmotic fragility test (OFT), autohemolysis test, acid glycerol lysis test, or pink test [2–5]. However, those tests have low sensitivity to detect cases with mild disease and are affected by several factors unrelated to the defects of red cell cytoskeleton [6]. To establish the diagnosis of HS in newborns by RBC morphology alone is unreliable because spherocytes and hyperdense RBCs may also be seen in normal newborns [7]. OFT may be normal in the presence of iron deficiency and obstructive jaundice, and in the recovery phase of aplastic crisis affected by increased reticulocytes [8]. Neonatal RBCs show different response to OFT than adult RBCs due to high hemoglobin (Hb) F concentration [9]. For the use in newborns, an incubated OFT is a recommended method [1, 9]. More specific tests such as the osmotic gradient ektacytometry and hypertonic cryohemolysis test are not widely available [10, 11].

Flow cytometric analysis with eosin-5-maleimide- (EMA-) labeled RBCs has shown a high sensitivity and specificity for diagnosing HS [12–14]. The fluorescent dye EMA binds to *ξ*-NH2 group of Lys-430 on band 3 protein which is a predominant protein in the RBC membrane [13]. The deficiency of band 3 protein results in a decrease of fluorescence intensity of EMA binding. Deficiencies of other RBC cytoskeletal proteins including band 4.2 and ankyrin also decrease fluorescence intensity because of the interaction between those proteins with band 3 [1]. The flow cytometric analysis with EMA-labeled RBCs has been recommended for diagnosis of HS in newborns [1, 15].

## 2. Case Presentation

A term male newborn with gestational age of 38 weeks was born by normal delivery. The birth weight was 2,350 g. His mother's blood type was O, Rh positive, and prenatal thalassemia screening test was negative. His mother and aunt has had mild hemolytic anemia and recently been diagnosed with HS. The patient was noted with jaundice at 20 hours of life. Physical examination showed jaundice without pallor and nonpalpable liver and spleen, while the rest of physical examination was unremarkable. He was investigated for the cause of neonatal hyperbilirubinemia. The peripheral blood smear showed spherocytes, crenated red cells, and polychromasia. The diagnosis of hereditary spherocytosis was suspected, and flow cytometry with EMA-labeled RBCs was performed to support the diagnosis.

## 3. Materials and Methods

Complete blood count (CBC), peripheral blood smear, and flow cytometric analysis with EMA-labeled RBCs of the patient and his parents were performed. The results are shown in Table 1. A healthy subject with normal hematological parameters and red blood cell morphology was investigated as a normal control. The patient's maximum total bilirubin level was 12.15 mg/dL at 20 hours of life. His blood type was O, Rh positive, and glucose-6-phosphate dehydrogenase (G-6-PD) assay by the fluorescent spot test showed normal result.

### 3.1. Flow Cytometric Analysis

The flow cytometric analysis with EMA-labeled RBCs was performed as previously described [14]. In brief, 100 *μ*L of whole blood in EDTA from each subject were washed twice with 1 mL of sterile phosphate buffered saline (PBS), pH 7.4, and centrifuged at 1,500 rounds per minute (rpm) for 5 minutes. Five *μ*L of red blood cells were incubated with 25 *μ*L EMA dye or PBS (as an unstained control) at room temperature in the dark for 1 hour. The cell suspension was centrifuged at 1,500 rpm for 1 minute. Then, the unbound dye was removed. The pellet with EMA-labeled RBCs was washed twice with 0.5 mL of sterile 0.5% bovine serum albumin (BSA) solution in PBS. Those EMA-labeled RBCs were suspended in 0.5 mL of 0.5% BSA in PBS. This cell suspension 100 *μ*L was added to 1.4 mL of 0.5% BSA in PBS solution for flow cytometric analysis. Mean fluorescence intensity (MFI) as mean of geometric mean (X-GMean) was determined for 10,000 events in FL1 channel of Beckman Coulter CyAn™ ADP High-speed Analyzer (Beckman Coulter, Fullerton, CA).

## 4. Results and Discussion

**Figure 1:**
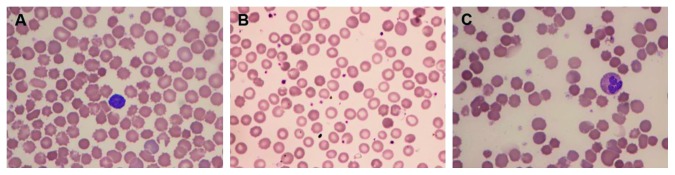
The peripheral blood smear (PBS) from the patient and his mother: (A) the patient at the age 2 days showing spherocytes, crenated red cells, and polychromasia; (B) the patient at the age 9 months showing spherocytes; (C) mother showing spherocytes and few crenated red cells.

**Figure 2:**
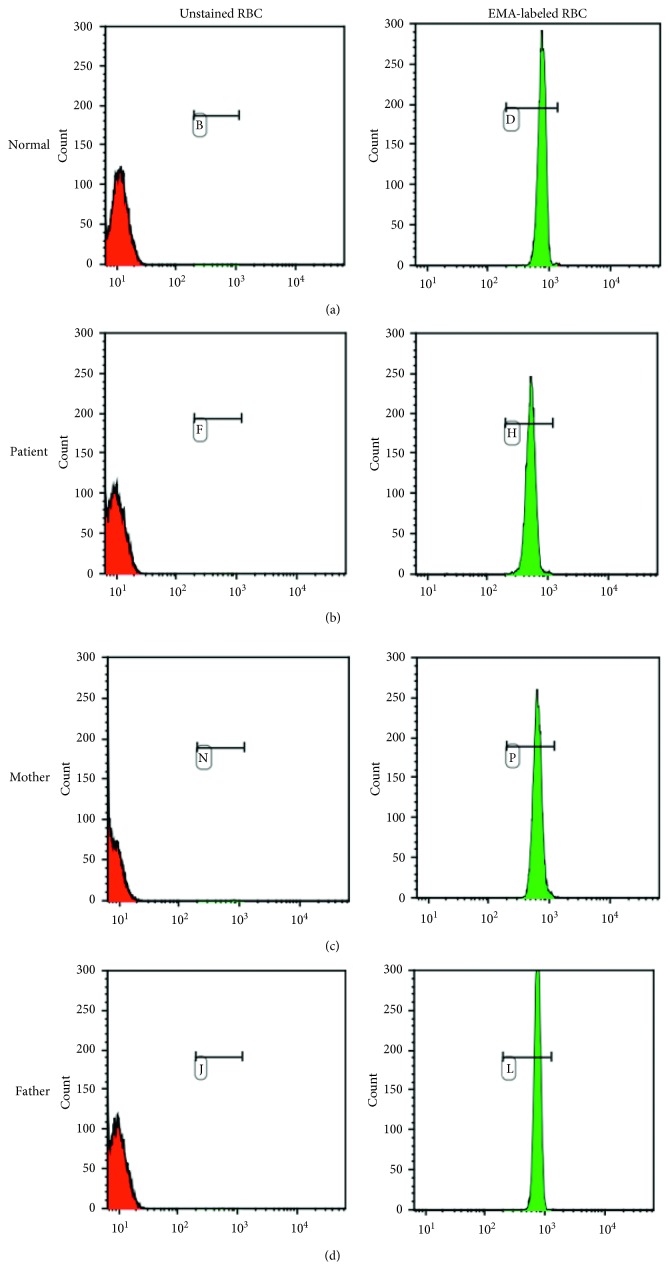
Fluorescence histogram of eosin-5-maleimide- (EMA-) labeled red blood cells of the patient and parents compared with a normal control. The unstained red blood cells and EMA-labeled red blood cells are shown in red and green, respectively.

Red blood cell parameters and flow cytometry results of the patient and parents are summarized in Table 1. The peripheral blood smears from the patient and his mother are as shown in Figure 1. The fluorescence histograms of EMA-labeled RBCs of the patient and parents compared with a normal control are shown in Figure 2. The patient had normal Hb level, while the mother had anemia. Spherocytes and crenated red cells were demonstrated in the patient and his mother. The MFI of the patient and mother was low in comparison with that of the father and control. The findings supported the diagnosis of HS in the patient and mother.

Indirect hyperbilirubinemia in newborns is caused by overproduction of bilirubin, impaired bilirubin uptake, or impaired bilirubin conjugation. Hemolysis is an important cause of increased bilirubin production resulting in neonatal indirect hyperbilirubinemia. The common causes of hemolysis in neonates include red blood cell membrane disorders, alpha-thalassemia disease, or G-6-PD deficiency. Impaired bilirubin conjugation could be found in G-6-PD deficiency or other inherited diseases, Gilbert syndrome, or Crigler–Najjar syndrome. HS was the most likely the cause of hyperbilirubinemia in our patient.

The patient was treated phototherapy for indirect hyperbilirubinemia for 2 days. He also received folic acid supplementation. RBC transfusion was not required. After phototherapy, the total bilirubin was reduced from 12.15 mg/dL at hour 20 of life to 9.53 mg/dL at 32 hours of life. The CBC of the last visit at the age of 9 months showed Hb of 11.1 g/dL, MCV of 66.7 fL, and MCHC of 32.9 g/dL with 15% spherocytes. His growth parameters and developmental milestones were normal for his age.

This report demonstrates the usefulness of flow cytometric analysis of EMA-labeled RBCs to diagnose HS in a newborn. The patient and his mother who had HS showed a decreased MFI and fluorescence ratio when compared with a normal control. The findings are consistent with the previous reports of HS in newborns [1, 16, 17]. Of note, in this case report, red cells from a healthy adult were used as a control. The guideline for a diagnosis of HS by EMA-flow cytometry in newborns suggests using red cells from healthy term newborns as controls [1, 7, 16]. However, two previous studies have shown that the EMA-flow cytometry results obtained from healthy newborns or neonatal cord blood red cells are not significantly different to those from adult red cells [18, 19]. These findings suggest that red cells from adults can be used as controls for a newborn study.

The 2011 guideline for diagnosis of HS recommends the flow cytometry with EMA-labeled RBCs for diagnosing HS [15]. This test is also useful for screening of atypical HS with mild severity. The test has been used in newborns with a high sensitivity (92.7–96.6%) and high specificity (99.1%) [14, 20]. The MFI ratio in adults with HS ranges from 59.1–90.1% [5]. The MFI in newborns with HS is distinctly lower than that in normal newborns [16]. The MFI ratios in our patient and his mother were 0.69 and 0.84, comparable to the MFI seen in HS patients.

The main limitation of the flow cytometric analysis with EMA-labeled RBCs is the need of fresh red cells. Alternatively, the red cells can be stored at 4°C for a maximum of 7 days [21–25]. The reproducibility of the results can also be affected by the dye stability and dye concentration [26]. The previous studies recommended reconstitution of the EMA dye every 6 months and storage at −20°C for 4 months [14] or −80°C for 6 months [26] in dark conditions. The test may be tested after a blood transfusion, which will show a double peak of fluorescence, indicating two red cells populations with normal fluorescence intensity and decreased fluorescence intensity [27].

The definite diagnosis of HS can be made by identification of the causative mutation. However, as the information of the involved genes and mutations causing HS remains limited, the current genetic identification technique is usually based on the whole-exome sequencing (WES) method which is costly and not widely available. Using flow cytometry with EMA-labeled RBCs provides a fast and practical diagnostic test which is helpful to identify HS as the cause of neonatal anemia and hyperbilirubinemia.

## 5. Conclusions

The careful examination of RBC morphology of the newborn and parents is the most crucial step of investigations of RBC disorders in newborns. EMA is a fluorescent dye that binds to band 3 protein. The deficiencies of band 3 and other adjacent proteins, ankyrin and band 4.2 which cause hereditary spherocytosis, result in a decrease of fluorescence intensity of EMA binding. Thus, the flow cytometry with EMA-labeled red blood cells is a practical and useful test for diagnosis of HS in newborn. The test is easily operated, rapid, and requires small amount of blood which is ideal to use in newborns.

## Figures and Tables

**Table 1:** Red blood cell parameters and flow cytometry results of the patient and parents.

Red blood cell parameters	Normal control	Patient	Mother	Father
Hemoglobin (g/dL)	12.2	17.1	9.6	15.9
Hematocrit (%)	38.4	54.1	31.6	51.1
Red blood cell count (×10^6^/mm^3^)	4.44	5.19	2.94	5.64
MCV (fL)	86.5	104.2	107.5	90.6
MCH (pg)	27.5	32.9	32.7	28.2
MCHC (g/dL)	31.8	31.6	30.4	31.1
RDW (%)	13.1	18.4	24.5	14.8
Reticulocyte (%)	—	4.2	—	—
Fluorescence intensity (X-GMean)	755.92	519.31	636.37	748.07
Fluorescence ratio	1.00	0.69	0.84	0.99
